# Prevalence and determinants of controlled hypertension in a German population cohort

**DOI:** 10.1186/1471-2458-13-594

**Published:** 2013-06-19

**Authors:** Neeltje van den Berg, Claudia Meinke-Franze, Thomas Fiss, Sebastian E Baumeister, Wolfgang Hoffmann

**Affiliations:** 1 University Medicine Greifswald, Institute for Community Medicine Ellernholzstrasse 1-2, 17487, Greifswald, Germany; 2 German Center for Neurodegenerative Diseases, site Rostock/Greifswald, Ellernholzstrasse 1-2, 17487, Greifswald, Germany

**Keywords:** Hypertension, Antihypertensive treatment, Determinants of normotensive blood pressure, Study of Health in Pomerania (SHIP)

## Abstract

**Background:**

Data of the German population-based cohort SHIP (Study of Health in Pomerania) were analysed to examine treatment rates, antihypertensive substances prescribed, and the proportion of hypertensive study participants reaching target values for blood pressure as well as determinants.

**Methods:**

The study population was defined using baseline data of the cohort (collected between 1997 and 2001). Participants with blood pressure values ≥140/90 mmHg and/or antihypertensive medication with known hypertension and participants with risk-comorbidity (diabetes, stroke, angina pectoris, and/or myocardial infarction) and blood pressure values ≥130/80 mmHg were included. The analysis of treatment and target values was based on the 5-year follow-up of the cohort (collected between 2002 and 2006). Logistic regression was used to identify determinants for a normotensive blood pressure.

**Results:**

3278 SHIP-participants with hypertensive blood pressure values were included (mean age: 55.5 years; SD 13.6, range 21–80 years). The raw hypertension prevalence was 50.9% (N = 1761). 58.7% (N = 1074) of all hypertensive patients reported some form of antihypertensive treatment. Thereof 65.1% (N = 728) received combination therapy. Of the patients without risk-comorbidity, 42.1% (N = 489) reached their target blood pressure values at the time of the 5-year follow-up of the cohort. Of the patients with any risk-comorbidity this proportion was only 21.7% (N = 131). Significant determinants for reaching the target values were being female and having antihypertensive combination therapy. Increasing age, having risk-comorbidities, and obesity were negatively associated with reaching the target values.

**Conclusions:**

Both the proportion of participants receiving therapy and the number of participants reaching their target blood pressure values are very low. Combination therapy is associated with better blood pressure control as compared to mono therapy. However, even in the subgroup of hypertensive patients under combination therapy only 36% (both patients with and without comorbidity) reach target values.

## Background

In Germany, about half of all adults have high blood pressure values (≥140/90 mmHg), with increasing prevalence in the higher age groups and higher rates in the male population [1,2]. Compared to other countries, the prevalence in Germany is high: in the USA hypertension prevalence is about 28%, in Italy 38%, in the UK 42% [3,4]. Hypertension is a risk for several secondary disorders such as coronary heart disease, cardiac infarction, and stroke. All these diseases are associated with high mortality and together are the number one cause of death in western countries.

Although patients with hypertension have various treatment options, there are large discrepancies between the numbers of patients with known, treated and controlled hypertension [1,2].

The population representative German Health Survey from 1998 showed, that 23.1% of the population had a known hypertension. Population prevalence of treated hypertension was 18.6%. In only 23.8% of all patients with treated hypertension blood pressure was sufficiently controlled (blood pressure values <140/90 mmHg) [5]. The proportion of both treated and controlled hypertension increases with increasing age, females are more often treated and have controlled blood pressure values than males. These observations have been confirmed by different German regional cohort studies [1,2].

Most important goal of the treatment of hypertension patients is to reach normotensive blood pressure values to decrease the risk for secondary disorders. Reaching normotensive blood pressure values can therefore be considered as an indicator of the effectiveness of antihypertensive therapy. According to major guidelines targeted values are below 140/90 mmHg [6,7]. Although targeted values below 130/80 mmHg in the current guidelines are recommended for patients with risk-comorbidity (diabetes, stroke, coronary heart disease, and renal insufficiency), assessment of recent studies has shown that the evidence for this is unclear. Nevertheless, actual reappraisals of the guidelines recommend treatment goals below 130/80 mmHg for hypertension patients with risk-comorbidity for the time being [8,9].

Pharmacotherapy of hypertension adopts a stepwise approach. Medical treatment can start with a single antihypertensive drug (monotherapy), usually an inhibitor of the angiotensin converting enzyme (ACE). If the target blood pressure values are not reached, the dosage should be increased. If this doesn’t show effect, an alternative drug should be prescribed. If the target blood pressure values cannot be reached under monotherapy, a combination of drugs can be prescribed (combination therapy, defined as treatment with two or more antihypertensive drugs from different substance classes). In many cases, a combination therapy is needed for patients with more severe hypertension [6,7,10]. Although the evidence is ambiguous, early treatment still is recommended [8].

In this paper, we analysed:

1. The proportion of hypertensive study participants reaching their target blood pressure values in the population-representative German SHIP-cohort. Target blood pressure values are <140/90 mmHg, for patients with risk comorbidities < 130/80 mmHg.

2. The proportions of hypertensive study participants reaching their target blood pressure values, separate for study participants without antihypertensive drugs, with mono, and with combination therapy.

3. Possible determinants of reaching target blood pressure values.

## Methods

Data from the Study of Health in Pomerania (SHIP) were included in the analysis. SHIP is an observational population based cohort study of adult German residents conducted in the region of Western Pomerania in northeast Germany. The main goal of SHIP is the provision of population-based data for the epidemiological analysis of a broad range of diseases, risk factors, and health indicators. SHIP was planned and accompanied with support and advice from an external Data safety and Monitoring Committee (DSMC). All participants gave written informed consent. The study conforms to the principles of the Declaration of Helsinki, as reflected by an a priori approval of the Ethics Committee of the Board of Physicians Mecklenburg-Pomerania at the University of Greifswald.

6267 persons (age 20–79 years) with German citizenship were drawn from population registries in the region of Western Pomerania, selected persons received up to three written invitations, followed by a telephone call or home visit in the case of non-response. At the end, the response was 68.7% (N = 4308) [11,12].

For this analysis we used data from the baseline (SHIP-0, collected between October 1997 and May 2001) to define the study population of participants with hypertension (concrete inclusion criteria: see further below) and the 5-year-follow up (SHIP-1, collected between October 2002 and September 2006) to examine treatment rates and determinants for controlled hypertension. Lifetime prevalence was used to define risk-comorbidity at the time of SHIP-1 [11,12].

From the 3300 persons that participated both in the baseline study (SHIP-0) and in the 5-year-follow up of the SHIP-study (SHIP-1), 22 participants were excluded because of missing information about the possible presence of hypertension. The analyses were based on the data of 3278 participants, thereof 51.9% (N = 1700) were female.

Sociodemographic data as well as the presence of diagnoses and risk factors were assessed in standardized, computer-assisted interviews and questionnaires. Blood pressure measurements in SHIP-0 and SHIP-1 were carried out with an automated oscillometric device (HEM-750CP, Omron Corporation, Tokyo, Japan). The measurements were conducted on the right arm after a rest period of five minutes in a sitting position. Blood pressure was measured three times at an interval of three minutes by trained and certified personnel. The mean value of the second and third measurement was used in the analysis. To assess medication, the participants were asked to bring all medication, taken during the last seven days, with them to the examination. All drugs were classified according to the Anatomical Therapeutic Chemical (ATC) classification [13]. Combined medical preparations were separated and classified according to each of their active substances. Drugs were classified as antihypertensive according to the guidelines on treatment of arterial hypertension of the German Hypertension Society [7].

We included SHIP-0-participants with blood pressure values in need of treatment:

study participants with blood pressure values ≥140/90 mmHg and/or antihypertensive medication with known hypertension;

study participants with risk-comorbidity (diabetes, stroke, angina pectoris, and/or myocardial infarction) and blood pressure values ≥130/80 mmHg. Risk-comorbidities were assessed by asking the patients for physician-confirmed diagnoses and the assessment of medication [11].

The participants were allocated to the different categories of the WHO/ISH-classification. This classification has seven categories: optimal blood pressure (syst: <120 mmHg/diast: <80 mmHg), normal (syst: 120–129 mmHg/diast:80–84 mmHg), high-normal (syst: 130–139 mmHg/diast: 85–89 mmHg), mild hypertension (syst: 140–159 mmHg/diast: 90–99 mmHg), moderate hypertension (syst: 160–179 mmHg/diast: 100–109 mmHg), severe hypertension (syst: ≥180 mmHg/diast: ≥110 mmHg), and isolated systolic hypertension (syst: ≥140 mmHg/diast: <90 mmHg) [14].

Descriptive statistics were applied to calculate prevalences and treatment rates. To identify possible determinants for normotensive blood pressure values, two multivariate logistic regression models were applied. Subjects with missing values on any of the analytical variables (n = 12) were excluded. Independent variables were sex, age, risk-comorbidity, obesity, smoking status, household size, and antihypertensive drug therapy. The first model includes active substances with the indication hypertension. The second model includes the kind of therapy (no therapy against mono or combination therapy).

All calculations were conducted using sample-design-weights to adjust for different selection probabilities within the sample [11] by using the SURVEY procedures by SAS 9.2, SAS Institute Inc., Cary, NC, USA.

## Results

**Figure 1:**
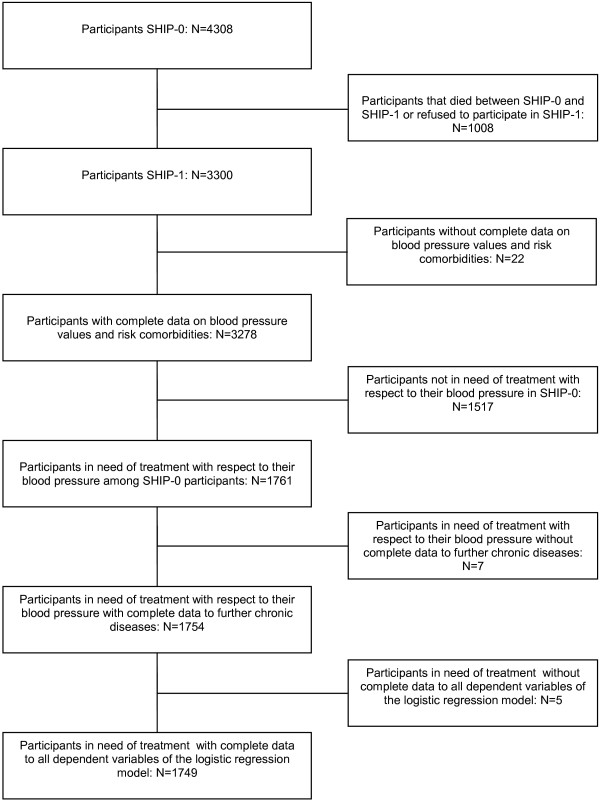
Flowchart of the numbers of patients included in the analyses.

The patients included in the different analyses are described in the flowchart in Figure 1.

### Prevalences and characteristics of hypertensive study participants

Applying the criteria described in the methods section, in SHIP-0, 1761 of 3278 included study participants had hypertension or blood pressure values in need of treatment (raw rate: 50.9%). The age-standardized hypertension prevalence in SHIP-0 was 39.4% (females 32.0%, males 47.3%). This prevalence can be considered as representative for the region Western Pomerania in which the SHIP cohort was recruited.

The mean age of the study participants with hypertension in SHIP-0 was 55.5 years (SD 13.6, range 21–80 years). The female study participants (N = 734) had a mean age of 56.7 years (SD 12.5, range 21–80 years), the male study participants (N = 1027) 54.6 years (SD 14.2, range 21–80 years).

The hypertension prevalences increase with higher age from 27.3% in the age group 25–34 years to 83.5% in the oldest age group.

Table 1 shows the number of participants, allocated to the different categories of the WHO/ISH-classification. The highest prevalences exist in the subcategories mild hypertension (33.6%) and isolated systolic hypertension (27.3%). Only 15.3% of the hypertensive participants and more females (22.4%) than males (9.7%) had blood pressures lower than 140/90 mmHg (optimal, normal, or high-normal blood pressure values).

**Table 1:** **Classification of blood pressure values (mmHg) of hypertensive patients in the SHIP-0 cohort**^**1**^**according to the WHO-guidelines [**[13]**]**

	**Females (N = 734)**		**Males (N = 1027)**		**Total (N = 1761)**	
	**N**	***% (95% CI***^***2***^***)***	**N**	***% (95% CI***^***2***^***)***	**N**	***% (95% CI***^***2***^***)***
**optimal**	23	*3.0*	9	*0.5*	32	*1.6*
syst: <120 mmHg/diast: <80 mmHg		*(1.7 – 4.3)*		*(0.1 – 0.9)*		*(1.0 – 2.2)*
**normal**	53	*6.8*	29	*2.5*	82	*4.4*
syst: 120–129 mmHg/diast: 80–84 mmHg		*(4.9 – 8.7)*		*(1.5 – 3.5)*		*(3.4 – 5.4)*
**high-normal**	94	*12.6*	78	*6.7*	172	*9.3*
syst: 130–139 mmHg/diast: 85–89 mmHg		*(10.1 – 15.1)*		*(5.1 – 8.3)*		*(7.9 – 10.7)*
**mild hypertension (grade 1)**	215	*31.3*	325	*35.4*	540	*33.6*
syst: 140–159 mmHg/diast: 90–99 mmHg		*(27.8 – 34.7)*		*(32.3 – 38.6)*		*(31.3 – 36.0)*
**moderate hypertension (grade 2)**	97	*13.7*	192	*20.5*	289	*17.5*
syst:160–179 mmHg/diast: 100–109 mmHg		*(11.2 – 16.3)*		*(17.7 – 23.3)*		*(15.6–19.5)*
**severe hypertension (grade 3)**	41	*5.1*	80	*7.1*	121	*6.3*
syst: ≥180 mmHg/diast: ≥110 mmHg		*(3.5 – 6.8)*		*(5.5 – 8.7)*		*(5.1 – 7.4)*
**isolated systolic hypertension**	211	*27.4*	314	*27.2*	525	*27.3*
syst: ≥140 mmHg/diast: <90 mmHg		*(24.1 – 30.8)*		*(24.4 – 30.3)*		*(25.1 – 29.5)*

^1^Study participants with blood pressure values ≥140/90 mmHg and/or antihypertensive medication with known hypertension and/or study participants with risk-comorbidity (diabetes, stroke, angina pectoris, and/or myocardial infarction) and blood pressure values ≥130/80 mmHg.

^2^*CI* confidence interval.

### Antihypertensive treatment

58.7% (N = 1074) of all hypertensive patients received some antihypertensive drug treatment. Gender differences are apparent: 67.5% of the female patients were treated, but only 51.8% of the males (p < 0.001). The proportion of treated hypertension patients increased with higher age.

Of all antihypertensive active substances, beta blockers were the most frequently prescribed substance class (63.7%, N = 671), ACE-inhibitors were taken by 52.4% (N = 586) of the treated hypertension patients. The next most prevalent substances were diuretics (41.1%, N = 475), and calcium channel blockers (30.6%, N = 347). Again there are differences between male and female patients: males take less beta blockers and more ACE-inhibitors as female patients (Table 2).

**Table 2:** **Distribution of antihypertensive substances in hypertension patients in the SHIP-0 cohort**^**1**^**to SHIP-1**

	**Females (N = 509)**		**Males (N = 565)**		**Total (N = 1074)**	
**Active agents**	**N**	***% (95% CI***^***2***^***)***	**N**	***% (95% CI***^***2***^***)***	**N**	***% (95% CI***^***2***^***)***
**Beta blockers**	335	*66.2*	336	*61.2*	671	*63.7*
		*(61.8 – 70.5)*		*(56.8 – 65.5)*		*(60.6 – 66.8)*
**ACE-inhibitors**	252	*49.6*	334	*55.3*	586	*52.4*
		*(45.0 – 54.2)*		*(50.9 – 59.7)*		*(49.2 – 55.6)*
**Diuretics**	242	*45.0*	233	*37.2*	475	*41.1*
		*(40.7 – 49.4)*		*(32.7 – 41.7)*		*(38.0 – 44.3)*
**Calcium channel blocker**	160	*28.9*	187	*32.3*	347	*30.6*
		*(24.9 – 32.9)*		*(28.0 – 36.7)*		*(27.6 – 33.6)*
**Angiotensin II receptor antagonists**	108	*20.1*	98	*17.3*	206	*18.7*
		*(16.4 – 23.8)*		*(13.9 – 20.6)*		*(16.2 – 21.2)*
**Antiadrenergic substances**^**3**^	22	*4.4*	34	*5.4*	56	*4.9*
		*(2.5 – 6.3)*		*(3.2 – 7.7)*		*(3.4 – 6.4)*
**Vasodilators**^**4**^	2	*0.3*	1	*0.2*	3	*0.2*
		*(0.0 – 0.8)*		*(0.0 – 0.5)*		*(0.0 – 0.5)*
**Other types**	-	*-*	1	*0.2*	1	*0.1*
				*(0.0 – 0.6)*		*(0.0 – 0.3)*

^1^Study participants with blood pressure values ≥140/90 mmHg and/or antihypertensive medication with known hypertension and/or study participants with risk-comorbidity (diabetes, stroke, angina pectoris, and/or myocardial infarction) and blood pressure values ≥130/80 mmHg.

^2^*CI* confidence interval.

^3^Both peripheral and central active antiadrenergic substances.

^4^Only with indication hypertension.

34.9% (N = 346) of the treated study participants received mono therapy, 65.1% combination therapy. Gender differences are small. The proportion of study participants with mono therapy decreased with increasing age from 81.8% (N = 10) in the youngest age group (25–34 years) to 20.1% (N = 50) in the oldest age group (≥ 75 years).

Most combinations comprised two substances (women: 43.4%, N = 151; men: 47.1%, N = 179), the most frequent combinations in females were ACE-inhibitors with diuretics and ACE-inhibitors with beta-blocker (prevalences of each combination 22.5%, N = 32). Male participants received most frequently combinations of ACE-inhibitor with beta-blocker (30.3%, N = 54) and ACE-inhibitor with diuretics (15.7%, N = 28). Also combinations of three substances were frequent (women: 37.9%, N = 132; men: 36.6%, N = 139).

### Analysis of determinants for normotensive blood pressure values

Table 3 shows the numbers and proportions of study participants with hypertension to the time of SHIP-0 with normotensive blood pressure values to the time of SHIP-1, separate for study participants with and without risk-comorbidities.

**Table 3:** **Number of hypertensive patients in the SHIP-0 cohort**^**1**^**reaching target blood pressure values to SHIP-1**

	**Female (N = 734)**		**Male (N = 1027)**		**Total (N = 1761)**	
	**N/N**_**tot**_	***% (95% CI***^***2***^***)***	**N/N**_**tot**_	***% (95% CI***^***2***^***)***	**N/N**_**tot**_	***% (95% CI***^***2***^***)***
**Patients without risk-comorbidity**^**3**^	(N = 487)		(N = 680)		(N = 1167)	
**no antihypertensive drugs**	74/174	*40.4*	152/380	*41.6*	226/554	*41.2*
		*(33.0 – 47.8)*		*(36.4 – 46.8)*		*(37.0 – 45.5)*
**mono therapy**	58/122	*46.5*	36/129	*31.4*	94/251	*39.1*
		*(37.3 – 55.6)*		*(22.4 – 40.2)*		*(32.7 – 45.5)*
**combination therapy**	92/191	*49.0*	77/171	*42.3*	169/362	*46.1*
		*(41.4 – 56.6)*		*(34.1 – 50.5)*		*(40.5 – 51.6)*
**total**	224/487	*45.1*	265/680	*39.8*	489/1167	*42.1*
		*(40.6 – 49.7)*		*(35.8 – 43.8)*		*(39.1 – 45.1)*
**Patients with risk-comorbidity**^**4**^	(N = 247)		(N = 347)		(N = 594)	
**no antihypertensive drugs**	17/51	*33.9*	13/82	*13.0*	30/133	*21.7*
		*(20.5 – 47.3)*		*(5.6 – 20.4)*		*(14.5 – 28.9)*
**mono therapy**	8/39	*21.8*	3/56	*5.8*	11/95	*12.5*
		*(7.7 – 35.9)*		*(0.0 – 12.5)*		*(5.2 – 19.8)*
**combination therapy**	37/157	*21.8*	53/209	*26.7*	90/366	*24.4*
		*(15.7 – 27.9)*		*(19.0 – 34.5)*		*(19.4 – 29.5)*
**total**	62/247	*24.5*	69/347	*19.6*	131/594	*21.7*
		*(19.0 – 29.9)*		*(14.3 – 24.8)*		*(17.9 – 25.5)*

^1^Study participants with blood pressure values ≥140/90 mmHg and/or antihypertensive medication with known hypertension and/or study participants with risk-comorbidity and blood pressure values ≥130/80 mmHg.

^2^*CI* confidence interval.

^3^Normotensive blood pressure: <140/90 mmHg.

^4^Risk-comorbidities: diabetes, stroke, angina pectoris, and/or myocardial infarction, normotensive blood pressure: <130/80 mmHg.

42.1% (N = 489) of the study participants without risk-comorbidity reached their target blood pressure values. The proportions of the female study participants show only slight differences between study participants with mono and with combination therapy (respectively 46.5% and 49.0%). Regarding male patients, there are larger differences between the patients with mono and combination therapy, 31.4% and 42.3%, respectively.

Lower target blood pressure values for patients with risk-comorbidities are reflected in lower proportions of normotensive patients (21.7%) in this subgroup. Regarding the patients with combination therapy, only small differences between males and females are apparent. Regarding the patients with mono therapy, only 5.8% of the male patients with risk-comorbidities reach normotensive blood pressure values (females: 21.8%).

Table 4 shows determinants of reaching target blood pressure values. Two regression models were calculated. In both models, risk-comorbidity (1. model OR = 0.39; CI = 0.30-0.52, 2. model OR = 0.40; CI = 0.30-0.53), obesity (1. model OR = 0.60; CI = 0.48-0.76, 2. model OR = 0.62; CI = 0.497-0.77), and increasing age (both models OR = 0.98; CI = 0.97-0.99) are negative determinants for achieving the target values. Female gender is a positive determinant (1. model OR = 1.29; CI = 1.03-1.63, 2. model OR = 1.33; CI = 1.05-1.67). In the first model, taking at least one beta blocker (OR = 1.28; CI = 1.01-1.64), has a positive influence on reaching target blood pressure values. In the second model, receiving antihypertensive combination therapy (OR = 1.54; CI = 1.16-2.05) is a positive determinant for achieving normotensive values.

**Table 4:** Multiple logistic regression analysis of determinants for normotensive blood pressure values

	**1. Model: Substances**	**2. Model: Therapy**
**Independent variables**	**OR**^**2**^**(95% CI**^**3**^**) **	**OR**^**2**^**(95% CI**^**3**^**)**
**Sex (ref. male)**		
**female**	**1.29 (1.03–1.63) (p = 0.030)**	**1.33 (1.05–1.67) (p = 0.016)**
**Age in years (cont. variable)**		
	**0.98 (0.97–0.99) (p = 0.001)**	**0.98 (0.97–0.99) (p = 0.001)**
**Risk-comorbidity (ref. no)**		
**yes**	**0.39 (0.30–0.52) (p < 0.0001)**	**0.40 (0.30–0.53) (p < 0.0001)**
**Obesity (ref. no)**		
**yes**	**0.60 (0.48–0.76) (p < 0.0001)**	**0.62 (0.49–0.77) (p < 0.0001)**
**Active smoker**^**4**^**(ref. no)**		
**yes**	0.97 (0.73–1.30) (p = 0.845)	0.97 (0.73–1.30) (p = 0.839)
**Household size (ref. > 1 pers.)**		
**1 person**	1.19 (0.91–1.57) (p = 0.208)	1.19 (0.91–1.57) (p = 0.206)
**Antihypertensive therapy(ref. no)**		
**mono therapy**	-	0.93 (0.69–1.26) (p = 0.650)
**combination therapy**	-	**1.54 (1.16–2.05) (p = 0.003)**
**Beta blocker (ref. no)**		
**yes**	**1.28 (1.01–1.64) (p = 0.044)**	-
**ACE-inhibitors (ref. no)**		
**yes**	1.04 (0.78–1.38) (p = 0.792)	-
**Diuretics (ref. no)**		
**yes**	1.32 (0.95–1.84) (p = 0.097)	-
**Calcium channel blocker (ref. no)**		
**yes**	1.03 (0.76–1.40) (p = 0.841)	-
**Angiotensin II receptor antag. (ref. no)**		
**yes**	1.13 (0.76–1.67) (p = 0.546)	-
**Antiadrenergic substances**^**5**^**(ref. no)**		
**yes**	1.16 (0.54–2.48) (p = 0.698)	-

^1^Study participants with blood pressure values ≥140/90 mmHg and/or antihypertensive medication with known hypertension and/or study participants with risk-comorbidity (diabetes, stroke, angina pectoris, and/or myocardial infarction) and blood pressure values ≥130/80 mmHg.

^2^*OR* Odds Ratio.

^3^*CI* confidence interval.

^4^Self reported smoking.

^5^Both peripheral and central active antiadrenergic substances.

Model 1 considers the active substances the patients received, model 2 considers the kind of therapy (mono or combination therapy). Included are all hypertensive patients^1^ to SHIP-0 with information on the independent variables to SHIP-1 (N = 1749 of in total 1761 hypertensive patients in the SHIP-0 cohort).

## Discussion

The analysis of the SHIP cohort provides detailed information about prevalences of hypertension, treatment characteristics and success of treatment of the population in the region Western Pomerania.

The results of the analyses show high prevalences of hypertension, both in the raw and age standardized rates.

Regarding medical treatment, differences between males and females were obvious, which is similar to the results of the German MONICA/KORA-studies [2]. Regarding the total group of hypertension patients, the proportion of treated patients increases with increasing age. Most treated patients received beta blockers, ACE-inhibitors, and diuretics. A majority of the treated patients (67.8%) received a combination therapy, and their proportion increases with age. Patients in the younger age groups received more often mono therapy. Patients with combination therapy received up to six different antihypertensive drugs. Since the study participants were asked to bring all their medication taken during then last seven days to the study centre, it is likely that a high proportion of documented drugs were actually taken by the SHIP-participants. However, there may be a risk of underreporting when the drugs can not be documented in the patients’ household [15].

Although the proportion of patients treated for hypertension is relatively high, only a small part of the patients reached their target blood pressure values. Positive determinants for reaching the target values were being female, receiving combination therapy and taking beta blockers. Negative determinants were increasing age, having risk-comorbidity (diabetes, stroke, angina pectoris, and/or myocardial infarction) and being obese.

The results of the analyses show that the management of hypertension is still a challenge, especially for patients with risk-comorbidity. A different result is shown in the Canadian Heart Health Surveys. Here, hypertension patients with cardiovascular or cerebrovascular diseases were more likely to reach their target values than patients without one of these diseases. The authors of the study suggest that this unusual result depends on a more intensive therapy to patients with a high risk or a better compliance of these patients [16]. Erkens et al. show in a study among hypertensive patients low persistence to medication, which leads to suboptimal treatment [17].

Nevertheless, non-adherence to antihypertensive medication may be a reason for not reaching the target blood pressure values. The results of a study on elderly patients of Turner et al. show that non-adherence leads to poorer blood pressure control in this patient group [18].

At the same time, non-adherence is a modifiable, patient-related factor [19] which belongs to a variety of drug related problems which affect treatment effectiveness [20].

Strenghts and limitations:

Strength of this analysis is the population based data base with highly standardized blood pressure measurement and structured assessment of antihypertensive medication.

We only documented drugs which were brought along by the patient to the study center. This increases data quality but may lead to some underreporting of drugs that were left at home. Several other drug-related-problems as the occurrence of drug-drug interaction may affect blood pressure level, too.

Another limitation is the absence of data to adherence, so the theory that treated hypertension patients with poor blood pressure values are less adherent can not be examined for the SHIP-participants.

## Conclusions

A high prevalence of hypertension and low rates of patients reaching target values of blood pressure values according to pertinent guidelines were shown in the German population based SHIP-cohort. These results support observations from other countries. Combination therapy is associated with better blood pressure control as compared to mono therapy. However, even in the subgroup of hypertensive patients under combination therapy only 36% (both patients with and without comorbidity) reach target values. Our data clearly show that patient- and provider-related intervention strategies need to be further developed, consequently implemented and prospectively evaluated.

## Competing interests

The authors declare that they have no competing interests.

## Authors’ contributions

NB, CM, TF, and WH participated in the design of the study. CM and SB performed statistical analysis. All authors participated in the interpretation of the results. NB drafted the manuscript. All authors read and approved the final manuscript.

## Pre-publication history

The pre-publication history for this paper can be accessed here:

http://www.biomedcentral.com/1471-2458/13/594/prepub
